# Acute Tentorial Subdural Hematoma Caused by Rupture of the Posterior Cerebral Artery after Minor Trauma—A Case Report

**DOI:** 10.3390/diagnostics10030175

**Published:** 2020-03-23

**Authors:** Urszula Maria Ciochon, Erik Gudmann Steuble Brandt, Trine Stavngaard

**Affiliations:** 1 Department of Diagnostic Radiology, Rigshospitalet, Blegdamsvej 9, 2100 Copenhagen, Denmark; erik.gudmann.steuble.brandt@regionh.dk (E.G.S.B.); trine.stavngaard@regionh.dk (T.S.); 2 Department of Radiology, Holbæk Hospital, Smedelundsgade 60, 4300 Holbæk, Denmark

**Keywords:** traumatic brain aneurysm, pseudoaneurysm, acute subdural hematoma, traumatic brain injury, endovascular coiling

## Abstract

Acute subdural hematoma (aSDH) is a common pathology encountered after head trauma. Only a minority of aSDHs have an arterial source. In this article, we report a case of aSDH originating from a traumatic pseudoaneurysm of the distal segment of posterior cerebral artery (PCA), diagnosed several days after the initial minor trauma and successfully treated with endovascular coiling. This case emphasizes the importance of searching for vascular pathology when the localization, severity or relapsing course of the intracranial hemorrhage does not fully correspond to the severity of initial trauma and when the bleeding has a delayed onset. Characteristics, diagnostics and treatment possibilities of traumatic cerebral aneurysms, an important cause of arterial aSDH, are described in the article.

## 1. Introduction

Acute subdural hematomas (aSDHs) often arise after head trauma due to a rupture of the cortical or bridging veins. Spontaneous aSDH of an arterial origin is on the other hand a rare clinical entity, which typically originates from branches of the middle cerebral artery (MCA) with truly spontaneous onset or following apparent minor trauma. A spontaneous aSDH of arterial origin arising from branches from the posterior cerebral artery (PCA) with no pre-existing aneurysms is exceptionally unusual, with only one case previously reported to our knowledge (Yasui 1995). We report a case of an initially misdiagnosed aSDH of arterial origin due to rupture of the P3/P4 segment of PCA after minor head trauma, which was eventually successfully treated by endovascular coiling. The objective of this case report is to highlight the possible causes of arterial aSDHs and the need to examine the patient for vascular pathologies if encountering a hemorrhage with an atypical clinical or radiological pattern.

## 2. Case Report

**Figure 1:**
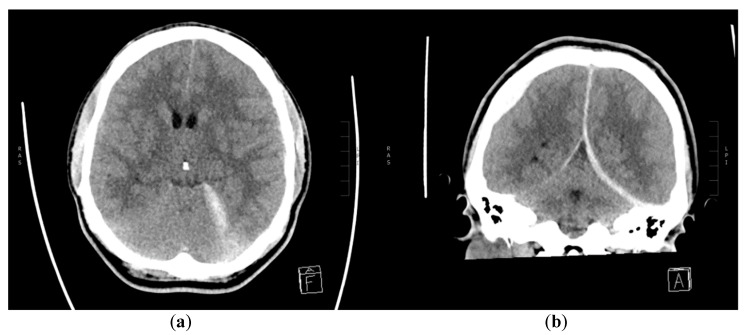
Non-contrast CT scanning of the brain on the day of first admission. (**a**) Axial and (**b**) coronal multiplanar reconstruction (MPR) showing left-sided aSDH along tentorium cerebelli and posterior interhemispheric fissure. The right-sided epidural hematoma (EDH) is also seen on image (**b**).

**Figure 2:**
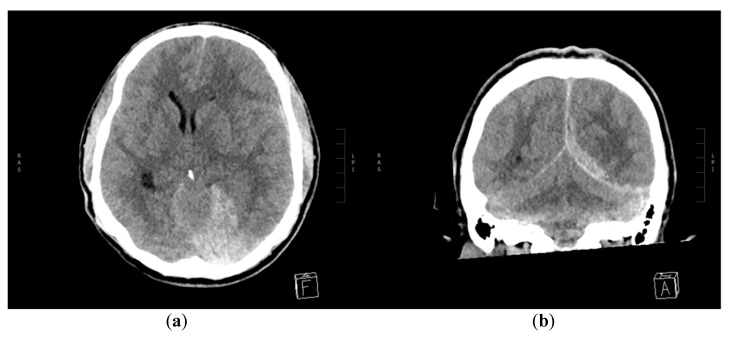
Control non-contrast CT of the brain upon readmission. (**a**) Axial and (**b**) coronal MPR reconstruction showing increased mass effect from the growing left acute subdural hematoma (aSDH) with midline shift to the right side, obliteration of quadrigeminal and both ambient cisterns, compression of the left lateral ventricle and dilatation of the right ventricular trigonum. The right-sided EDH is not shown.

**Figure 3:**
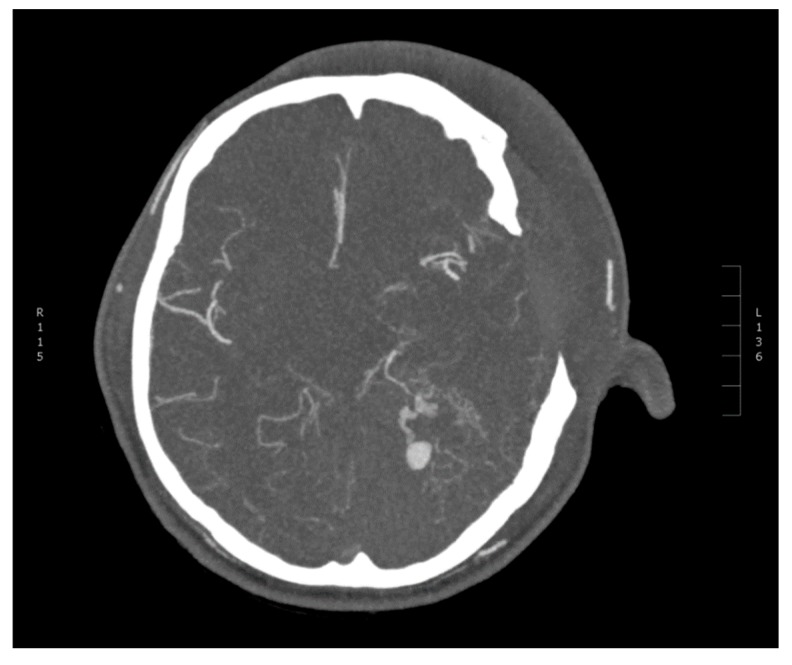
CT angiography of cerebral arteries. Axial maximum intensity projection (MIP) showing a spot sign close to the left tentorium cerebelli.

**Figure 4:**
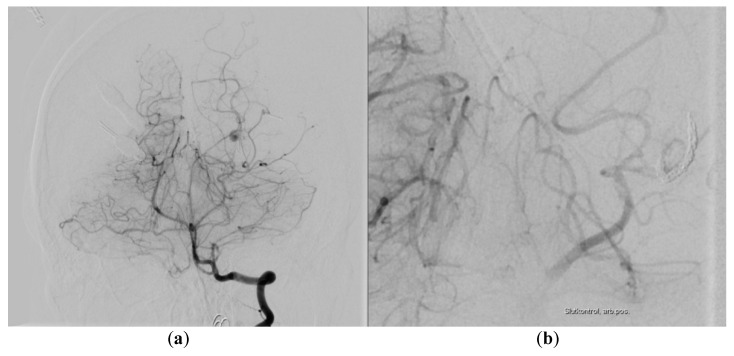
Digital subtraction angiography (DSA): (**a**) Injection in the right vertebral artery, anteroposterior (AP) view showing a pseudoaneurysm on a distal posterior cerebral artery (PCA) branch. (**b**) Zoomed post-embolization control AP image showing coils in the parent PCA segment and no filling of the pseudoaneurysm.

A 30-year-old male without past medical history reported himself to the emergency ward after a minor crash on a bicycle against a runner earlier that day. He was not wearing a helmet and he was not under the influence of alcohol or drugs. There was no loss of consciousness and his Glasgow Coma Scale (GCS) was 15. He had a minor excoriation on the forehead, pain in his left elbow and right wrist, where fractures were subsequently excluded. Initially, no computed tomography (CT) scan of his head was performed due to unremarkable neurological examination. However, before leaving the emergency ward, his headache worsened (scored as 6 on the Visual Analogue Scale) and his GCS decreased to 14 points (with eye opening on command). He became somnolent, there was no extremity paresis or cranial nerve abnormality and his pupils were equal in size, but with a slightly delayed response to light. A CT scan was therefore performed, which showed a small right-sided parietal epidural hematoma (EDH) and small posterior left-sided tentorial and posterior interhemispheric subdural hematoma (SDH) with only a minor local mass effect (Figure 1). No subarachnoidal (SAH), intracerebral hemorrhage (ICH) or cranial fractures were seen. He was admitted to the neurosurgical ward and was observed there for three days. His headache, GCS 14 and otherwise unremarkable neurological status persisted, but the control CT-scans showed a slight decrease in both EDH and SDH. Afterwards, he was transferred to a local hospital where his condition was unchanged until day 9 after the initial trauma, where he had two generalized tonic-clonic seizures. His GCS declined to 3, all extremities became tonic with periodical extensions and the pupils were dilated and unresponsive to light stimuli, with left pupil being larger than right. He was intubated, sedated and readmitted to the neurosurgical ward with a CT scan showing increasing size of the left-sided aSDH in addition to bilateral supratentorial edema (Figure 2) interpreted as a result of the seizures. An external ventricular drain (EVD) and an intracranial pressure gauge were placed with initial intracranial pressure (ICP) measuring 96 mmHg. Despite attempts to decrease the ICP (head elevation, hypertonic saline infusion, muscle relaxation and moderate hyperventilation), the ICP values remained high and unstable (fluctuating between 27 and 120 mmHg). Thus, on the following day, a hemicraniectomy had to be performed, resulting in a postoperative ICP of 25 mmHg. The patient still had a GCS of 3, no response to painful stimuli, and equal and unresponsive pupils. The intensive medical measures were continued, aiming for an ICP value below 20 mmHg, which could not be achieved. CT angiography (CTA) was performed to exclude the diagnosis of central venous thrombosis as a cause of the raised ICP. The CTA showed large left-sided tentorial and falcine aSDH with a spot sign on the angiography (Figure 3). This was unfortunately interpreted as blood of varying age. Partial re-evacuation of the SDH was performed on day 13 because of the hard texture of the hematoma and the difficult access as a result of the prominent brain edema. However, the ICP decreased to 19–20 mmHg with continued intensive medical measures, yet the patient still had GCS 3 with equal and unresponsive pupils and no reaction to painful stimuli. During day 15, after an abrupt, unprovoked 10–15 minute long episode of ICP measuring 40 mmHg and otherwise unchanged neurological status, control CT showed a reformed aSDH on the same site. The previous CT angiography was re-evaluated and a diagnostic digital subtraction angiography (DSA) was performed during day 16 revealing active extravasation intro subdural space from a pseudoaneurysm on a distal branch of the left PCA equivalent to the P3/P4 segment (Figure 4). No additional bleeding source was seen after contrast injection into both internal carotids. Good cerebral perfusion was seen, though slightly affected by the aSDH and sequelae after previous craniectomy. An 80 cm AXS Infinity LS Long Sheath (Stryker Neurovascular) was introduced, a Sofia 5F (Microvention) guiding catheter was placed in the left PCA and an Excelsior SL-10 (Stryker Neurovascular) microcathether was advanced with Traxcess guidewire (Microvention) to the left PCA, where microinjections showed active extravasation originating from a pseudoaneurysm of the lateral branch of distal PCA. The microcatheter was then advanced past the bleeding site and the distal branch including the pseudoaneurysm was then embolized with seven coils (Stryker). The extravasation stopped immediately and no retrograde contrast filling of the pseudoaneurysm was seen on control DSA. The aSDH was then definitely evacuated by repeated craniectomy immediately after the endovascular procedures. Afterwards, the SDH and edema slowly subsided, the ICP values remained below 20 mmHg, but the endovascular coiling was complicated by infarction of the major part of the left PCA territory. The patient’s GCS and neurological status did not improve until nine days after coiling, where his pupils began to react to light stimuli and brainstem reflexes with spontaneous respiration also appeared. Sedatives were subsequently discontinued. On day 21 after coiling, his GCS improved to four points (with eye opening in reaction to painful stimuli, continued tetraparesis and no verbal response) and slowly increased to GCS 11 during the subsequent days (eye response, verbal response and motor response). One month after endovascular occlusion, the patient was transferred for a highly specialized neurorehabilitation with swallowing training, joint mobilization, weight-bearing exercises during tilt table inclination with mobilization of the neuraxis and cognitive training. Persistent peripheral muscle contractures were treated with systemic baclofen and intramuscular injections of the botulinum toxin. After six months, the patient was in a relatively good physical condition, although confused (GCS 14), suffering from cognitive difficulties and resting right-sided hemiparesis, but able to follow simple verbal commands and capable of movement with assistance from another person (four points on the modified Rankin Scale). This research has obtained the patient’s consent.

## 3. Discussion

Acute subdural hematoma (aSDH) can occur in a broad range of head traumas as a result of the rupture of cortical bridging veins or surface veins, with blood accumulation between dura mater and arachnoid membranes. It complicates about 11% of mild to moderate traumatic brain injuries (TBIs) requiring hospitalization, and about 20% of severe TBIs [1]. Up to 20–30% of aSDHs can originate from the rupture of small cortical arteries [1,2] from stretching or torque injury [3]. Most frequently, aSDH is related to a history of TBI, but cases of spontaneous aSDH without clear history of TBI are also reported, such as from aneurysm rupture into the subdural space, bleeding from vascular malformations, dural metastases, vascular meningiomas, spontaneous intracranial hypotension, infections or from the use of antithrombotic medication [1].

Infrequently, aSDH may result from the rupture of a traumatic intracranial aneurysm (TIA) [4]. This aneurysm type is very rare, representing below 1% of all intracranial aneurysms [5] and carrying a high rate of morbidity and up to 50% mortality [6]. Typically, TIAs result from severe TBI, with only a few cases of minor brain injury reported [7]. Their presentation can be insidious and delayed, resulting in hemorrhage several hours to several weeks, or even several years after the initial trauma [8,9]. Most of these aneurysms arise close to the skull base or near the falx, where the artery can be easily stretched and damaged during the head trauma moment [9]. The most common location of TIAs are anterior circulation vessels, with involvement of the supraclinoid internal carotid artery (ICA) in about 50% of cases [10], followed by peripheral branches from MCA, as well as pericallosal artery [11]. Initial CT angiography may be negative for vascular injury, followed by bleeding with various delay after the TBI; therefore, it has been recommended to repeat DSA 1–2 weeks after TBI in patients suspected for vascular injury [12]. In particular, a non-contrast CT scan on admission with a volume of SAH larger than expected for the severity of TBI, as well as hematomas near the skull base or in parafalcine structures should advocate additional imaging for possible TIA [10]. Some TIAs can origin from a dissection with intramural hematoma or vessel outpouching, resulting in true, false or mixed type aneurysm [10,13]. Our patient’s history and imaging findings point toward a traumatic false aneurysm (pseudoaneurysm) on the peripheral left PCA with repeated rebleeding exclusively into the subdural space during several days after TBI. As the patient was not examined with a vascular study until almost two weeks after TBI, it cannot be reliably concluded whether the aneurysm appeared at the time of TBI or some days afterwards. The unfortunate misinterpretation of findings on the CTA caused further delay in performing diagnostic DSA and definitive treatment of the lesion. Moreover, aSDH is a common finding after TBI and typically does not warrant additional vascular imaging, especially after minor trauma as in our patient, as vascular pathologies constitute a minority of etiologies that can result in pure aSDH without corresponding SAH or ICH.

In the case of aSDH originating from PCA described by Yasui et al. [14], there was no history of trauma, presenting symptoms were in the form of sudden headache and vomiting, and the patient was known to have liver dysfunction due to chronic alcohol abuse. His CT showed bilateral aSDH in the posterior interhemispheric fissure, and DSA 6 h after the symptom onset showed contrast extravasation from the distal branches of right PCA and left anterior cerebral artery (ACA). The left-sided aSDH was evacuated surgically and the bleeding from peripheral PCA branch on the left medial occipital lobe was ceased by bipolar coagulation. No aneurysm, vascular malformation or malignancy was reported. The authors suggested that the PCA might have been torn by a sudden brain movement caused by a sharp head movement that was not severe enough to be recognized as a minor trauma. Arterial hypertension was also considered as another possible cause for this aSDH. The patient’s chronic liver insufficiency might also have contributed with possible bleeding diathesis due to clotting factors deficiency.

Because of the rarity of TIAs, there are no randomized controlled trials providing treatment recommendations. Our patient’s pseudoaneurysm was successfully treated with coils, though complicated by infarction of the left PCA territory. Unfortunately, some TIAs can be difficult to be treated endovascularly because of strenuous catheterizing to reach the peripherally located aneurysm, risking perforation or dissection of the parent vessel and distal infarction. Additionally, coiling or clipping of the fragile pseudoaneurysm can easily lead to intraprocedural aneurysm rupture. Other treatment possibilities include endovascular or surgical parent vessel sacrifice as well as stenting or placement of flow diverters [10,15]. Some stents can be successfully deployed even in vessels with a diameter smaller than 2 mm (Neuroform—Atlas (Stryker Neurovascular) [16]; Leo Baby (Balt, Montmorency, France); LVIS (Microvention) [17,18]). In exceptional cases, TIA can thrombose and occlude spontaneously; such aneurysms are indicated by very late contrast filling on the diagnostic DSA [10]. However, due to very high mortality and morbidity after rebleeding occlusion of TIAs is to be recommended at the earliest possibility after establishing the diagnosis.

## 4. Conclusions

Some acute subdural hematomas can have an arterial origin. Traumatic brain aneurysms constitute a possible etiology and can result in delayed rebleeding with a very high mortality rate. Repeated vascular studies should be advised shortly after traumatic brain injury in patients suspected for vascular damage and the aneurysm should be occluded early after the diagnosis.
